# Impact of the Familiar Environment in 11–14-Year-Old Minors’ Mental Health

**DOI:** 10.3390/ijerph15071314

**Published:** 2018-06-23

**Authors:** Benito León-del-Barco, Fernando Fajardo-Bullón, Santiago Mendo-Lázaro, Irina Rasskin-Gutman, Damián Iglesias-Gallego

**Affiliations:** 1 Department of Psychology, Teacher Training College, University of Extremadura, 10071 Cáceres, Spain; bleon@unex.es (B.L.-d.B.); smendo@unex.es (S.M-L.); irasskin@unex.es (I.R.-G.); 2 Department of Psychology, Faculty of Education and Psychology, University of Extremadura, 06006 Badajoz, Spain; 3 Department of Didactics of Music, Plastic and Body Expression. Teacher Training College, University of Extremadura, 10071 Cáceres, Spain; diglesia@unex.es

**Keywords:** mental health, preadolescence, adolescence, family, parental affection, parental rejection

## Abstract

The analysis of the mental health in children under 14 years has become a research topic of global interest where the family can be a key factor for protection or risk against mental health problems. With this work, we intend to determine, employing binary logistic regression analysis, whether parental acceptance-rejection perceived by boys and girls can predict their mental health. Seven hundred sixty-two students participated, the average age was 12.23 years; 53.8% (n = 410) girls and 46.2% (n = 352) boys. We have used the Strengths and Difficulties Questionnaire (SDQ), self-reported version and the Affection Scale children version (EA-H) for parental acceptance-rejection to assess mental health. The odds ratio (OR) of the logistic models reports that there is a greater probability of having mental health problems in boys and girls when they perceive that they are highly criticized and rejected by their parents. With our work, we highlight the importance of the environment and family affection on mental health. The perception of the children about the rejection, aversion, and criticism of their parents constitutes a risk factor in the manifestation of mental health problems.

## 1. Introduction

### 1.1. Mental Health and Family Variables

Mental health problems in children and adolescents raise a great social concern because they are associated with disability, suffering, functional deterioration, and carry a great economic cost for public health throughout the world [1,2]. Overall, it is estimated that between 10% and 20% of children suffer from mental health problems [3]. Other studies, such as the Spanish National Health Survey 2006, carried out with the Strengths and Difficulties Questionnaire (SDQ) [4], indicated that between 19.2% and 26.6% of the Spanish children and adolescents aged 4 to 15 years were at risk of suffering from mental health problems [5]. Furthermore, the review carried out by Polanczyk, which included 41 studies conducted in 27 countries from all regions of the world, found that the prevalence of mental disorders in children and adolescents was 13.4% [6]. The analysis of the mental health of children under 14 years old has become a topic of research of global interest where the family can be a key factor for their protection or risk at the time they face mental health problems [7]. In this sense, there is a significant association between suicide attempts and relationship problems among the adolescents [8], and family could be a main source of these relationship problems. Parents are the main source of socialization and development of every person from an early age. In the general population the influence of the family context on the psychosocial development of children is indisputable; parents are one of the most powerful influences in the lives of their children [9].

The main studies that have used the Strengths and Difficulties Questionnaire (SDQ) to relate family variables, emotional disorders, and behavioral problems as indicators of mental health, have analyzed different factors such as education level, social class, family type, employment status, and parental health. Regarding the education level, the results show that there is a strong association between the education level of parents and the children’s mental health informed by parents [10,11,12,13,14]. In this regard, the children of mothers with a primary education level had worse mental health than the children of mothers with university education level [15]. According to this, when a mother obtains a high level of education, it reduces the probability of children having mental health problems [16].

For the studies that have related occupational class of parents with mental health, three social classes are frequently used: high, medium, and low [5]. The results show that those jobs of the primary wage earner with lower categories are those most related to the presence of mental health problems in children [17], where parental rejection was more prevalent among parents with little education and a low family socio-economic level [18]. On the contrary, it is the families with jobs corresponding to the middle and the most privileged classes that become a higher protective factor against the presence of mental health problems in the minors than those linked to disadvantaged classes [12,13,15,17]. In general, those children with families with poorer socioeconomic status have worse physical and mental health than those with a higher socioeconomic level [14,19,20,21]. As socioeconomic status decreases, there is an increment of family stress and the use of corporal punishment; this, in turn, can predict behavior problems in minors [22,23].

Other variables that have been shown to be significant for the minor's mental health have been the type of single-parent family [12,13,15], as well as the fact that some family members, or all of them, are unemployed [16]. Likewise, the influence of poor mental health of the parents and the health of the mother has been analyzed and there is evidence to show that the mental health of the parents, especially the health of the mother, are related to the mental well-being of their children [24,25].

### 1.2. The Present Study 

Some studies have analyzed family relationships from the point of view of family violence, either between parents [26,27] or from parents to children [28]. In this sense, the “Interpersonal Acceptance-Rejection theory” (IPARTheory) [29] analyzes the influence of parents’ affections on their children and how these perceptions or experiences can affect the cognitive and socio-emotional adjustment of children [9]. According to Rohner [29], the parental acceptance-rejection implies a continuum. At one end would be parents who show their children love and affection both verbally and physically, at the other end we would find parents who feel aversion towards their children, criticize them, and reject them [30]. This theory has been internationally accepted with research in more than 22 different countries [31]. Some remarkable results show how affection and communication prevent misbehavior in children and stimulate positive development [32]. In this sense, the positive affectivity of parents on children constitutes a preventative requirement for mental health [33], with rejection being a risk factor in the appearance of psychopathologies [34], violent behavior, and poor emotional decoding abilities [35]. 

On the other hand, investigations such as Muris, Meesters, Morren, and Moorman [36], Repetti, Taylor, and Seeman [37], and Steinberg [38], confirm that inadequate levels of affection and support, as well as the predominance of aggression and rejection towards the children, are related to the manifestation of behavioral problems of aggressiveness, hostility, and delinquency. We know that a lack of affection from the mother, together with the rejection of the father, can be related to a greater probability of being an aggressor in situations of bullying [39], but we do not know the direct relationship with the minor's mental health. Some studies indicate that rejection by both parents is a mediating factor between parental depression and minor’s mental health problems, influencing psychosocial difficulties [40]. In turn, children’s perception of a positive affection from their parents seems to be a preventive factor for good mental health [41].

It is known that in the first years of adolescence development, minors are more exposed to behavioral or relational problems, learning to manage and regulate their behavior as they move towards a middle or late stage of adolescence [42]. Although as the children grow they perceive a decrease in support and involvement in both parents, as well as a weaker control and supervision are noticed [43], various studies from the perspective of parental acceptance-rejection have already shown how age does not influence the psychological adjustment produced in situations of rejection by both parents [44]. Despite the fact that multiple factors (such as individual, developmental, family, social, and contextual factors) may affect the parental function, the focus in this research has been on the type of family relationship.

The conceptions of parents about education, interpersonal relationships, and parenting styles are determining factors in the development of children. More studies are needed to analyze the child-to-parent relationships of rejection and affection, and its relation to the minor’s mental health. With this work, we aimed to determine, by means of a binary logistic regression analysis, if the acceptance-rejection perceived by the children related to their parents can predict their mental health. Why are we interested in the perception of children about the acceptance-rejection of their parents? First, there is a low coincidence between the opinions of parents and children about parental practices. Parents usually have their own perception of their parental practices, sometimes biased by a social desirability. On the other hand, the perception of adolescent children is less biased and is more objective, thus it can be an important predictor of their responses, indeed, more so than the perceptions of their parents [45,46]. For this reason, we also use the Strengths and Difficulties Questionnaire (SDQ) in the self-reported version to evaluate mental health.

## 2. Materials and Methods 

### 2.1. Sample

The sample was made up of 762 students. The mean age was 12.23 years (SD = 1.122, range 11–14); 53.8% (n = 410) girls and 46.2% (n = 352) boys. The number of participants was determined from the number of students enrolled in Primary Education and Compulsory Secondary Education (ESO) in public and private schools in the Community of Extremadura (Spain) during the 2016-2017 academic year, considering a sampling error of 3% and a confidence level of 95.5%. The selection of the sample was made through a multistage sampling by conglomerates and random selection of the groups in the centers that had several classrooms per grade, 5th and 6th of Primary Education and 1st and 2nd of Compulsory Secondary Education. Cluster sampling was carried out by randomly selecting four schools. Regarding the distribution by course of our participants, 22% were in the 5th grade of Primary Education (10–11-year-olds), 23.1% in the 6th grade of Primary Education (11–12-year-olds), 26.3% were in the 1st grade of Compulsory Secondary Education (12–13-year-olds), and 28.6% were in the 2nd grade of Compulsory Secondary Education (13–14-year-olds).

This age interval presents typical aspects in terms of psychological development that must be taken into account. Many studies confirm that the endocrine changes that begin in late-childhood, and that continuous through puberty, increases the risk of health problems, especially those concerned with emotional and behavior control [47,48]. These changes, along with their interaction with the social environment in which the child develops, are key for understanding the adolescent adjustment in terms of mental health. Parenting behavior also changes during this stage of lifespan [49,50] along with the increment of family conflicts due to a renegotiation of positions within the family [49,51].

### 2.2. Data Collection Instruments

Strengths and Difficulties Questionnaire, SDQ [4]. This questionnaire has proved to be an excellent screening tool compared to other more classic ones such as the Child Behavior Checklist [52,53]. The SDQ has been validated internationally [54] and has been used in many international studies for the measurement of mental health. There are three versions of the SDQ: one for parents, one for teachers, and another for self-report that is advisable after the age of 11. The content and structure of the SDQ has been developed using as a reference the childhood mental health disorders described by the Diagnostic and Statistical Manual of Mental Disorders of the American Psychiatric Association. [55,56]. The SDQ-self-reported version is a brief instrument, excellent for the screening of mental health disorders in children, taking into account the last 6 months, with an internal consistency highlighted in all its scales, both internationally [57,58] and in its Spanish version [59,60]. It consists of 25 items divided into five dimensions or subscales (1. Emotional Symptoms, 2. Behavioral Problems, 3. Peer Problems, 4. Hyperactivity, and 5. Prosocial Behavior). Each of the subscales is valued through five items. The answer format is of the Likert type with 3 degrees (0 = No, nothing, 1 = Sometimes, and 2 = Yes, always). The total score in difficulties is obtained by adding the four scales without considering the prosocial scale. 

For community samples, it is recommended to group the items of the Behavioral Problems subscale and the items of the Hyperactivity subscale in a new scale called Externalizing Problems [55]. In the same way, the items of the subscale Emotional Symptoms are added with the items of the subscale Peer Problems to create the Internalizing Problems scale [54]. Both the total score of the SDQ and the scores of the different subscales and scales are classified into three categories: Normal, Borderline, and Abnormal. In the original scale [4], the limit of the Abnormal category corresponds to the score that delimits the top 10% of cases (Percentile ≥ 90), while the Borderline category corresponds to 10% of cases between the 80th percentile and 90th percentile. The overall reliability of the scale presents a Cronbach's alpha (α) of 0.66 and Composite Reliability (CR) of 0.80, the scale of Internalizing Problems an α of 0.57 and CR of 0.72, and the scale of Externalizing Problems a α of 0.45 and CR of 0.76.

Affection Scale children version, EA-H [61]. It consists of two factors, each of them with 10 items that are presented in a Likert scale format, with five degrees of frequency that represent a continuum from Never to Always. The first factor called Affection-Communication evaluates the perception that children have of the affection, interest, and communication that their parents (Father-Mother) express towards them: “It comforts me when I am sad”, “It accepts me as I am”, “He is affectionate to me”. The first factor has an α of 0.85 and CR FC of 0.86 for the parent mode and for the mother modality an α = 0.82 and CR = 0.84. The second factor called Critical-Rejection evaluates the criticism, the rejection, and the lack of parental trust (Father-Mother) towards their children: “What I do seems wrong to him”, “He is upset when I am at home”, “He would like it to be different”. It has α = 0.82 and CR = 0.83 for the parent mode and α = 0.77 and CR = 0.79 for the mother modality.

### 2.3. Procedure

The procedure followed for collecting data was the administration of the questionnaires by classroom group. In the first place, we contacted the educational centers to explain the objectives of the study and request authorization for the completion of the questionnaires. We followed the ethical guidelines of the American Psychological Association [62] regarding the informed consent of the parents, due to participants’ being underage. Likewise, anonymity in the answers, the confidentiality of the obtained data, and its exclusive use for research purposes was assured. The administration of the questionnaires was done during school hours, it took around 40 min in an adequate climate and without distractions. This study was approved by the Bioethics and Biosafety Committee of the University of Extremadura (NO. 0063/2018).

### 2.4. Data Analysis

The statistical analysis carried out included analysis of reliability of the instruments used, measure of association *odds ratio* (OR), and binary logistic regression using the SPSS “enter method”. Prior to these analyses, another analysis of missing data was performed with the variables included in the models analyzed. As we have mentioned, the analyses were performed with the statistical package SPSS version 21.0 (IBM Corp., Armonk, NY, USA) for personal computer (PC).

## 3. Results

Our main objective is to determine, through a binary logistic regression analysis, whether the acceptance-rejection perceived by boys and girls of their parents can predict their mental health. However, by initially using the OR ratio statistics tools, we want to analyze the association between participants’ gender and mental health. In order to achieve this, the total scores of the SDQ (Mental Health Problems), the Internalizing Problems scale and the Externalizing Problems scale were used as dependent variables. These variables were transformed into dichotomous variables from the 80th percentile (*p* ≥ 80 = “Suffering Problems”; *p* < 80 = “No suffering Problems”). In the category of “Having Mental Health Problems, Having Internalizing Problems and Having Externalizing Problems” were grouped the subjects of the Abnormal and Borderline categories of the original scale [4]. 

As we can see in Table 1, there is a significant association between the variable “gender” and the *SDQ Total Score* and the *Externalizing Problems*. Regarding the association with the *SDQ Total Score*, the following data were obtained: OR = 2.199, the limits of the ratios, 95% confidence, did not contain the unit, and the χ^2^ = 18.421 confirmed the significance of these results (*p* < 0.001). Thereby, being a boy is a risk as far as the presence of mental health problems is concerned. The probability of having mental health problems is 2.19 times higher in boys than in girls. Also, regarding the association with the *Externalizing Problems* variable, an OR = 2.484 was obtained and both the limits of the ratios (1.746–3.535) and the χ^2^ = 26.456 confirmed the significance of the results (*p* < 0.001). The probability of having *Externalizing Problems* is almost 2.5 times higher in boys than in girls.

Regarding our main objective, in order to confirm whether children’s perception of affection, communication, criticism, and rejection by their parents (father or mother) can significantly predict the mental health of their children, a binary logistic regression analysis was carried out. The dichotomous variables of the total score of the SDQ (Mental Health Problems), of the *Internalizing* Problems scale, and of the Externalizing Problems scale were used as dependent variables and as predictor variables, the four factors of the EA-H (Affection and Father communication, Affection and Mother communication, Criticism and rejection Father, Criticism and rejection Mother) were grouped as dichotomous variables (Normal = 0, High = 1) from the 80th percentile (*p* < 80 = 0, *p* ≥ 80 = 1).

Three predictive models are created by gender (girls and boys) (Table 2). The predictive model for the total score of the SDQ (*Mental Health Problems*) allows a correct estimation of 85.8% of the cases (χ^2^ = 27.706(4), *p* < 0.001) in girls (Cox & Snell’s R^2^ = 0.066; Nagelkerke’s R^2^ = 0.118) and of 76.7% of the cases (χ^2^ = 25.7634), *p* < 0.001) in Boys (Cox & Snell’s R^2^ = 0.071; Nagelkerke’s R^2^ = 0.103). The model for *Internalizing Problems* allows a correct estimation of 81.4% of the cases (χ^2^ = 33.145(4), *p* < 0.001) in girls (Cox & Snell’s R^2^ = 0.078, Nagelkerke’s R^2^ = 0.125) and of 78.4% of cases (χ^2^ = 18.908), *p* = 0.001) in boys (Cox & Snell’s R^2^ = 0.052, Nagelkerke’s R^2^ = 0.080). The model for *Externalizing Problems* is not significant in the case of girls (χ^2^ = 4.475(4), *p* = 0.3461) and allows a correct estimation of 72.7% of the cases (χ^2^ = 27.611(4), *p* < 0.001) in the case of the boys (Cox & Snell’s R^2^ = 0.075; Nagelkerke’s R^2^ = 0.106). 

The OR of the logistic models report that: (1) the probability of having mental health problems is 5.7 times higher in girls who perceive that they are highly criticized and rejected by their father and 3 times higher in boys who perceive that they are highly criticized and rejected by their mothers; (2) the probability of having internal problems is 6 times higher in girls who perceive that they are highly criticized by their fathers; (3) boys perceive that they are highly criticized or rejected by their mothers are 2.2 and 2.5 times more likely to have internal and external problems, respectively.

## 4. Discussion

The results confirm that being a boy is a risk factor for the presence of mental health problems. The probability of having mental health problems is 2.19 times higher in boys than in girls. Also, the probability of having *Externalizing Problems* (Behavioral and Hyperactivity) is almost 2.5 times higher in boys than in girls. These results are consistent with a large majority of research that, independently of the version of SDQ used (Parents, Teachers, or Self-Reported), confirm that males obtain a higher score in external symptoms, such as behavior problems and hyperactivity [63,64,65,66]. A wider range of studies that support these same results can be observed in the work carried out by Ortuño-Sierra that includes an international review [42] and in other academic literature about meta-analysis of transcultural studies [67]. 

Regarding our main objective, the ORs of the logistic models report in a general way that there is a greater probability of having mental health problems and manifest internal and external symptoms in boys who perceive that they are highly criticized and rejected by their mothers. Girls who perceive that they are highly criticized by their fathers are more likely to have mental health problems and internal symptoms. The rejection, aversion, and criticism that children perceived by their parents constitutes a risk factor in the manifestation of mental health problems. These results coincide with other recent studies with a Spanish population of similar characteristics [44,68,69] and with Repetti's studies [37] that relate parental styles characterized by a predominance of aggression and rejection towards children with the manifestation of mental health problems and behavioral problems of aggression, hostility, and delinquency. Numerous studies highlight how a negative emotional attitude characterized by lack of affection and communication increases the risk of manifesting behavioral and health problems [46,70] regardless of whether the child or the parents report [32].

Based on the results obtained in our research, we asked ourselves some questions. One of them was about why the rejection of fathers is a risk factor for girls and the maternal rejection a risk factor for boys? Regarding this issue, various investigations show that the influence exerted by fathers and mothers in the upbringing of their children is different.

In general, social learning theories expected that the behavior of the father would be more determinant for the boys, since it is a learning role model as could be the mother for the daughters [71]. However, the results are uneven. In general, girls feel the influence of both parents, while boys perceive a greater influence of mothers. Likewise, parents are more likely to overprotect their daughters than their sons, and the parenting factors associated to the father appear as predictors of negative behavior in daughters [46,72,73]. In this sense, some studies indicate that difficulties with the father at 13–14 years produce a higher cost in the mental health of girls than they produce in boys [74], or that the father’s preference to spend more time with the son than with the daughter may influence the lower presence of negative symptoms in the son [75], but not in the daughters [71]. Taking into account the differences related to gender, the situations of maternal rejection in minors seems to have a greater emotional impact in sons than in daughters, according to stated memory when they become adults [76]. Linking with the type of symptomatology analyzed above, other studies support our results with a differentiation depending on whose rejection, paternal or maternal, is concerned. Specifically, the contribution of paternal rejection tends to develop emotional disorders like negative self-esteem, negative self-adequacy, emotional unresponsiveness, and emotional instability. All of which are related to internalizing disorders that appear more often in girls. In a different direction, the contribution of maternal rejection is higher regarding the externalizing dimensions of hostility and dependence, more characteristic in boys [44]. In this sense, it is interesting to observe that when there is a greater participation and involvement of the father, the externalizing and internalizing symptomatology of the minors is diminished in both sexes [31,71,77]. Due to this, it would be interesting to incorporate the measurement of this variable in future investigations.

Another question we asked was about why acceptance, love, and communication of parents are not factors of protection against mental health problems? In our case, it seems that there are no differences in terms of affection and communication between parents and their children, boys and girls. This may be due to the greater affective autonomy present in the developmental stage in which they are beginning as adolescents [44]. However, the rejection of the father worsens mental health problems in the daughters, and the rejection of the mother worsens mental health problems in the sons. Coinciding with other studies [34], the rejection factor is a highly determinant factor, in comparison with acceptance, for the appearance of psychological and behavioral problems in minors [76], demonstrating its influence at a transcultural level [78]. This invalidating environment could lead to borderline personality disorders [79] and various, internalized and/or externalized alterations in the children, such as depression, suicidal behavior, anxiety, aggressiveness, and hostility [31,76]. However, other studies have suggested that parental acceptance is a more relevant factor for boys than for girls, and even that father´s rejection is the most influential in boys and girls [31]. Finally, we can observe how our results complement the international studies on parental acceptance-rejection [80], providing a greater influence of the rejection of fathers and mothers in children of the opposite sex, between 11 and 14 years old. 

### Study Limitations and Future Directions. 

The study has several limitations, such as the use of self-reports by participants both for the evaluation of mental health problems and parental acceptance-rejection. It is important to point out that, although the content and structure of the SDQ has been developed using as a reference the childhood mental health disorders described by the Diagnostic and Statistical Manual of Mental Disorders (DSM-IV) [55,56], and it has proven to be a good predictor of disorders reported in the International Classification of Diseases (ICD-10) [81], the SDQ is a mental health screening tool, not a determinant by itself for clinical diagnosis [7]. The questionnaire employed in the present study measures child's self-perception of affection, communication, criticism, and rejection of their parents. The use of self-reported questionnaires, such as the SDQ, for measuring health conditions in national surveys is widely used [82]; however, the findings may be biased if there is a differential recall based on mental health problems. Despite the fact that the SDQ refers to the last 6 months, and the EA-H to the moment when the child is completing the questionnaire, it is important to take into account that there may be a bias on recall known as “effort after meaning” [83,84], through which the child can justify his or her symptoms based on past experiences [85]. Thus, we consider that it would be important to have other informants in addition to those directly involved. In this sense, teachers have a privileged situation to analyze minor’s mental health problems since they can observe and evaluate them in the classroom in daily situations, and they can establish comparisons with other children of the same developmental level. Likewise, within a bidirectional model of parent-child relationships, it would be convenient to evaluate mental health and acceptance-rejection from the point of view of parents. On the other hand, the results should be analyzed with caution when extrapolating them to other countries, especially non-Western ones, because of the cultural influence [78]. Finally, this study did not assess other potential confounding factors which contribute to depression and family problems in children and adolescents including obesity [86], chronic diseases such as asthma [87] and dermatitis [88] leading to negative impact on family [89], online isolation due to excessive internet use [90], and father involvement [77].

## 5. Conclusions

The conclusions of this study strengthen the evidence of how the probability of having mental health problems, measured by the SDQ-*self-reported version* in Spanish minors, is more than double in boys than that in girls, this difference can be even greater when it comes to *externalizing symptoms* (Behavior Problems and Hyperactivity). However, no differences are obtained in the *internalizing symptoms* according to the gender of the Spanish minors. 

On the other hand, the probability of suffering mental health problems increases in girls 5.7 times when they perceive *criticism and rejection* of the father. In the same line, the probability of suffering mental health problems in boys triples when they perceive *criticism and rejection* of the mother. No significant differences appear when they suffer this rejection by the parental figure of their same sex.

More specifically, it is observed that girls who perceive *criticism and rejection* of their father, are 6 times more likely to suffer mental health problems of the *internalizing* type. In the case of boys who perceive *criticism and rejection* by the mother, they are twice as likely to have mental health problems in both symptomatology, internalizing, and externalizing.

Finally, we are aware of how mental health problems in children and adolescents cause great social concern and entail a great economic cost for public health throughout the world. With our work, we have highlighted the importance of the environment and family affection on mental health. The rejection, aversion, and criticism perceived by the children from their parents constitutes a risk factor in the manifestation of mental health problems. People who have been emotionally rejected tend in turn to reject any affective approach of other people, and favor a climate of lack of affection and rejection towards others [24]. Understanding the factors associated with mental health problems in children and adolescents can facilitate early identification of children at risk and the implementation of prevention programs [14]. Likewise, it would be advisable to design intervention and training programs with families that can improve relations between parents and children [46]. We also believe in parent training oriented to discover if the origins of their children’s problems can be found in their own behavior. This training would increase the awareness of parents and the use of their skills and competences. Parent-training programs are a way to promote their development, they improve family relationships and reinforce the sense of satisfaction and competence of parents regarding their tasks and parental responsibilities. Finally, new research seems to be necessary that can provide possible solutions, both from the point of view of intervention with the parents—to prevent their children from having a tendency to social rejection and acceptance difficulties—as well as from prevention through direct work with the minors [76].

## Figures and Tables

**Table 1:** Odds ratio (OR) depending on the different dependent variables studied (*Mental Health Problems, Internalizing Problems, Externalizing Problems*) and gender.

SDQ	Gender	% Problems	Rate	χ^2^	*p*	OR	95% CI	
Mental Health Problems	Girls	14.2	0.382	18.421	<0.001	2.199	1.527	3.166
	Boys	26.7	0.618					
Internalizing Problems	Girls	19.1	0.494	1.495	0.221	1.244	0.876	1.767
	Boys	22.7	0.506					
Externalizing Problems	Girls	15.1	0.365	26.456	<0.001	2.484	1.746	3.535
	Boys	30.7	0.635					

SDQ = Strengths and Difficulties Questionnaire. Rate = proportion of different gender categories with presence of mental health problems.

**Table 2:** Results of the logistic regression analysis for the prediction of *Mental Health Problems* (Total SDQ) *Internalizing Problems* and *Externalizing Problems* in girls and boys.

SDQ		Factors ^a^	B	SE	Wald	df	Sig.	Exp(B)	95% CI	
Total	Girls	Affection and Communication Father	−0.569	0.529	1.155	1	0.282	0.566	0.201	1.597
		Criticism and Rejection Father	1.738	0.432	16.212	1	0.000	5.684	2.439	13.243
		Affection and Communication Mother	0.110	0.494	0.050	1	0.824	1.116	0.424	2.941
		Criticism and Rejection Mother	−0.367	0.469	0.614	1	0.433	0.692	0.276	1.736
		Constant	−2.060	0.218	89.557	1	0.000	0.127		
	Boys	Affection and Communication Father	0.342	0.418	0.669	1	0.413	1.407	0.621	3.192
		Criticism and Rejection Father	0.156	0.421	0.138	1	0.710	1.169	0.513	2.667
		Affection and Communication Mother	0.791	0.388	4.152	1	0.052	2.205	1.031	4.716
		Criticism and Rejection Mother	1.083	0.395	7.530	1	0.006	2.952	1.363	6.397
		Constant	−1.575	0.180	77.012	1	0.000	0.207		
Internalizing Problems	Girls	Affection and Communication Father	−0.693	0.454	2.324	1	0.127	0.500	0.205	1.219
		Criticism and Rejection Father	1.805	0.412	19.213	1	0.000	6.078	2.712	13.622
		Affection and Communication Mother	0.399	0.424	0.888	1	0.346	1.491	0.650	3.419
		Criticism and Rejection Mother	−0.426	0.447	0.908	1	0.341	0.653	0.272	1.569
		Constant	−1.716	0.192	79.916	1	0.000	0.180		
	Boys	Affection and Communication Father	0.668	0.432	2.391	1	0.122	1.950	0.836	4.547
		Criticism and Rejection Father	0.245	0.434	0.320	1	0.572	1.278	0.546	2.991
		Affection and Communication Mother	0.194	0.422	0.212	1	0.645	1.214	0.531	2.776
		Criticism and Rejection Mother	0.920	0.409	5.059	1	0.025	2.509	1.126	5.591
		Constant	−1.715	0.187	83.654	1	0.001	0.180		
Externalizing Problems	Girls	Affection and Communication Father	−0.195	0.437	0.199	1	0.656	0.823	0.350	1.937
		Criticism and Rejection Father	0.244	0.460	0.282	1	0.595	1.277	0.519	3.142
		Affection and Communication Mother	0.478	0.425	1.265	1	0.261	1.612	0.701	3.707
		Criticism and Rejection Mother	0.538	0.465	1.343	1	0.247	1.713	0.689	4.258
		Constant	−1.947	0.206	89.078	1	0.000	0.143		
	Boys	Affection and Communication Father	0.542	0.407	1.773	1	0.183	1.719	0.774	3.818
		Criticism and Rejection Father	0.402	0.408	0.967	1	0.325	1.494	0.671	3.328
		Affection and Communication Mother	0.452	0.385	1.381	1	0.240	1.572	0.739	3.341
		Criticism and Rejection Mother	0.946	0.382	6.141	1	0.013	2.575	1.219	5.442
		Constant	−1.354	0.168	64.868	1	0.000	0.258		

^a^ Reference category is ‘no’; B = Unstandardized beta coefficient; SE = standard error; df= Degrees of freedom.
